# Acute kidney injury in imported *Plasmodium falciparum* malaria

**DOI:** 10.1186/s12936-015-1057-9

**Published:** 2015-12-24

**Authors:** Liese C. Koopmans, Marlies E. van Wolfswinkel, Dennis A. Hesselink, Ewout J. Hoorn, Rob Koelewijn, Jaap J. van Hellemond, Perry J. J. van Genderen

**Affiliations:** Institute for Tropical Diseases, Harbour Hospital, Haringvliet 2, 3011 TD Rotterdam, The Netherlands; Department of Medical Microbiology and Infectious Diseases, Erasmus MC, Rotterdam, The Netherlands; Department of Internal Medicine, Erasmus MC, Rotterdam, The Netherlands

**Keywords:** Malaria, Falciparum, Kidney injury, Renal failure, Renal replacement therapy

## Abstract

**Background:**

Acute kidney injury (AKI) is a known complication of malaria, and is reported to occur in up to 40 % of adult patients with a severe *Plasmodium falciparum* infection in endemic regions. To gain insight in the incidence and risk factors of AKI in imported *P. falciparum* malaria, a retrospective analysis was performed on a large cohort of mostly non-immune patients with imported *P. falciparum* malaria. Aiming to include not only severe but also milder forms of renal failure, the KDIGO criteria were used to define AKI.

**Methods:**

Clinical and laboratory data from 485 consecutive cases of imported *P. falciparum* malaria were extracted from the Rotterdam Malaria Cohort database. Acute kidney injury (AKI) was defined using the KDIGO criteria. Univariate and multivariate logistic regression analyses were used to identify risk factors for AKI.

**Results:**

AKI was seen in 39 (8 %) of all patients and in 23 (38 %) of the 61 patients with severe malaria. Eight patients eventually needed renal replacement therapy (RRT); seven of them already had AKI at presentation. Higher age, higher leucocyte count and thrombocytopaenia were independently-associated with AKI but their positive predictive values were relatively poor.

**Conclusion:**

AKI was found to be a common complication in adults with imported *P. falciparum* necessitating RRT in only a small minority of patients. The use of the KDIGO staging allows early recognition of a decline in renal function.

**Electronic supplementary material:**

The online version of this article (doi:10.1186/s12936-015-1057-9) contains supplementary material, which is available to authorized users.

## Background

Acute kidney injury (AKI) is associated with a risk of chronic kidney disease and high mortality [1–3]. The clinical course of *Plasmodium falciparum* malaria may be complicated by AKI, contributing to the high mortality rate of severe malaria [4]. The pathogenesis of AKI in malaria is still not clearly understood. Blockage of renal microcirculation due to sequestration of infected erythrocytes, immune-mediated glomerular injury and volume depletion are some of the proposed hypotheses [4, 5]. The main histopathological finding in malaria-associated AKI is acute tubular necrosis (ATN) with reports of interstitial nephritis and glomerulonephritis [4–7]. In malaria-endemic regions, AKI can occur in up to 40 % of adult patients with severe *P. falciparum* malaria, and it is associated with a mortality as high as 75 % when renal replacement therapy (RRT) is not started in time [8–11]. In non-immune travellers with severe *P. falciparum* infection, AKI is reported to occur in 34 to 52 % of cases [12–15]. In these studies, AKI is usually defined according to the WHO criteria for severe malaria, in which the creatinine threshold is preset at 265 μmol/L [13–15]. Using this relatively high threshold, less severe forms of renal failure are not taken into account. The present study, a retrospective analysis on a large cohort of patients with both severe and non-severe imported *P. falciparum* malaria, was designed to include these milder forms of renal failure in the analysis. To detect these, the criteria set by the Kidney Disease: Improving Global Outcomes (KDIGO) Acute Kidney Injury Work Group, in which the dynamics of the GFR changes are taken into account, were used to define renal failure [16]. The study further aimed to determine which parameters are associated with the development of AKI.

## Methods

### Patients

The Harbour Hospital is a 161-bed general hospital, located in Rotterdam, The Netherlands. It also harbours the Institute for Tropical Diseases, which serves as a national referral centre. The Rotterdam Malaria Cohort consists of all patients diagnosed with malaria at the Institute for Tropical Diseases since 1998. Of all patients, anonymized demographic, clinical and laboratory data are routinely collected and stored in an electronic database. For the present study, data from patients with imported *P. falciparum* malaria who entered the Rotterdam Malaria Cohort before January 1st, 2015 were analysed retrospectively. At that moment, the Rotterdam Malaria Cohort comprised 690 cases of imported malaria, of which 491 were caused by *P. falciparum*. Six cases were excluded because of missing creatinine values at initial presentation, leaving 485 patients for the analysis. The six excluded cases had a favourable outcome and did not require RRT.

As this is a retrospective study of patient files, ethical approval was not necessary, as stated in the Medical Research involving Human Subjects Act (WMO).

### Laboratory investigations

All laboratory data were measured on admission with routine procedures, as described before [17]. The standard procedure to diagnose malaria comprised a Quantitative Buffy Coat (QBC) analysis, a rapid diagnostic test (RDT) for malaria antigens (Binax NOW^®^ Malaria Test Binax, Inc. Maine, USA), and thick and thin blood smears using freshly collected blood specimens from finger pricks. The RDT and the QBC analysis were performed according to the manufacturer’s instructions. QBC capillaries were examined independently by two technicians by microscopic analysis of two complete rows of the region between the bottom of the capillary and the polynuclear leukocyte layer using an Olympus BX-60 fluorescence microscope equipped with UV-filter, 50× objective and 12.5× oculars (total magnification 625×).

### Definitions

#### Acute kidney injury

Acute kidney injury (AKI) was defined using the KDIGO criteria, which include three stages of progressive renal dysfunction (see Additional file 1) [16]. Since patients may present with renal dysfunction without a known baseline serum creatinine, a classification based on a change of creatinine or estimated glomerular filtration rate (eGFR) from baseline poses a significant limitation. To circumvent this problem, the following method, which was previously described as a suitable method by the ADQI Group [18], was used: in patients without previously measured creatinine levels, a normal premorbid renal function was assumed. A baseline creatinine level was then calculated, using the four-variable modification of diet in renal disease (MDRD) formula with an assumed normal eGFR. These calculated baseline values were used to estimate the change of renal function during infection (see Additional file 2).

#### Severe malaria

Patients were classified as having severe *P. falciparum* malaria if they met the recently updated World Health Organization (WHO) criteria for severe malaria on admission or during hospitalization (see Additional file 3) [19].

#### Immunity to malaria

Adult immigrants from a malaria-endemic country living in The Netherlands were considered partially immune, as they had likely been exposed to *P. falciparum* during childhood. Patients who had been born and raised and were still living in a malaria-endemic area at the time of diagnosis were presumed semi-immune. However, given the low number of semi-immune persons in the Rotterdam Malaria Cohort, they were grouped with partially immune individuals for the statistical analysis. All other patients were considered non-immune.

### Statistical analysis

All data were extracted from the original database. For statistical analysis IBM Statistical Package for the Social Sciences (SPSS) version 22 (IBM Inc., Chicago, IL, USA) was used. Differences between the groups “AKI” and “no AKI” were analysed using Fisher’s exact test or Chi square test for nominal variables. For continuous variables the unpaired two sample *t* test or the Mann–Whitney *U* test was used. Normality of distribution of variables was tested with the Kolmogorov–Smirnov test. Univariate and multivariate logistic regression analyses were used to identify risk factors for AKI in malaria at initial presentation. Based on the univariate analysis, variables with a *P* value <0.10 were included in the multivariate logistic regression. The details of the univariate and multivariate regression analysis are available as supplementary files (see Additional files 4 and 5). For the variables that were significant in the multivariate analysis, the diagnostic performance for detecting KDIGO-defined AKI was evaluated, using the odds ratio, sensitivity, specificity, negative and positive predictive value at the optimal cut off point (determined using the Youden’s index) and the area under the receiver operating characteristics curve (AUROC). All reported *P* values are two-tailed, and *P* values less than 0.05 were considered statistically significant.

## Results

### Patient characteristics and incidence of AKI

The general characteristics on admission of the 485 included patients are shown in Table 1. Fifty-nine patients were treated as outpatient or finished their treatment at home, all other patients were treated as inpatients. None of the patients had serum creatinine levels measured in The Harbour Hospital prior to admission; the premorbid creatinine levels were, therefore, calculated according to the described method to subsequently apply the KDIGO classification. AKI, defined as a KDIGO stage 1, 2 or 3, was seen in 39 (8 %) of all 485 patients with imported falciparum malaria and in 23 (38 %) of the 61 patients with severe malaria. Immunity differed significantly between the groups with and without AKI, with non-immunes being over-represented in the AKI group. The use of potentially nephrotoxic co-medication (non-steroidal anti-inflammatory drugs (NSAIDs), ACE-inhibitors, diuretics or lithium) did not differ significantly between groups. Pulse rate was significantly higher in the AKI group and the systolic blood pressure was significantly lower in comparison to patients without AKI. Apart from creatinine and urea, also leukocytes, C-reactive protein (CRP), creatinine, total bilirubin, lactate, lactate dehydrogenase (LDH), alanine transaminase (ALAT), aspartate transaminase (ASAT) and parasite load levels were all significantly higher in the AKI group, while thrombocytes and serum sodium levels were significantly lower. There was no significant difference in the number of patients with circulatory shock between the two groups (three in the AKI group versus 13 in the no-AKI group. Severe malaria was significantly more common in the AKI group, as might be anticipated since AKI is one of the defining criteria for severe malaria (see Additional file 3). There were two deaths, both in the AKI group.

**Table 1:** General characteristics on admission

	AKI n = 39	No AKI n = 446	*P* value
*Demographic findings*			
Age (years)	47 (26–70)	39 (4–78)	<0.001
Male gender	27 (69)	325 (73)	0.708
Ethnicity			0.078
Caucasian	24 (62)	189 (42)	0.028
African	11 (28)	218 (49)	0.018
Asian	2 (5)	14 (3)	0.374
Other	2 (5)	22 (5)	1.000
Adequate prophylaxis use	0 (0)	42 (9)	0.052
Symptoms ≥8 days	16 (41)	111 (25)	0.055
Immunity			0.038
Non-immune	28 (74)	218 (53)	0.007
Partially immune	10 (26)	188 (45)	0.060
Semi-immune	0 (0)	9 (2)	1.000
Nephrotoxic co-medication*	5 (20)	24 (5)	0.073
*Clinical findings*			
Temperature (°C)	38.5 (1.3)	38.4 (1.3)	0.852^
Systolic blood pressure (mmHg)	114 (21.3)	123 (18.3)	0.017^
Pulse rate (beats/min)	104 (20.1)	94 (17.3)	0.003^
Glasgow Coma Scale	15 (5–15)	15 (9–15)	<0.001
*Laboratory findings*			
Haemoglobin (mmol/L)	8.1 (1.8)	8.3 (1.3)	0.929^
Leucocytes (×10^9^/L)	7.5 (3.2)	5.2 (2.0)	<0.001^
Thrombocytes (×10^9^/L)	29 (2–188)	91(3–385)	<0.001
CRP (mg/L)	185 (16–476)	91 (1–363)	<0.001
Creatinine (µmol/L)	166 (77–1081)	93 (39–175)	<0.001
Urea (mmol/L)	13.8 (4.1–55.8)	5.0 (1.5–33.6)	<0.001
Sodium (mmol/L)	131 (6.2)	135 (4.0)	<0.001^
Potassium (mmol/L)	3.7 (0.6)	3.8 (0.4)	0.422^
Bilirubin total (µmol/L)	52 (10–416)	22 (3–304)	<0.001
ASAT (U/L)	96 (9–394)	32 (9–326)	<0.001
ALAT (U/L)	68 (12–655)	36 (3–265)	<0.001
LDH (U/L)	488 (127–2297)	268 (103–1833)	<0.001
Lactate (mmol/L)	3.0 (1.0–6.2)	1.4 (0–5.5)	<0.001
Parasitaemia (parasites/µL)	128,000 (240–1,380,600)	8400 (2–784,000)	<0.001
*Concomitant bacterial infection*	5 (13)	9 (2)	0.003
Treatment			<0.001
Quinine iv	19 (49)	54 (12)	<0.001
Artesunate iv	13 (33)	27 (6)	<0.001
Atovaquone/proguanil	4 (10)	279 (63)	<0.001
Halofantrine	1 (3)	54 (12)	0.108
Artemether/lumefantrine	0 (0)	4 (1)	1.000
Unknown	2 (5)	28 (6)	1.000
Outcome			
Severe malaria (WHO 2014)	29 (74)	32 (11)	<0.001
Renal replacement therapy	8 (21)	0 (0)	<0.001
Death	2 (5)	0 (0)	0.006

*P* values <0.05 are considered significant

* Self-reported use of non-steroidal anti-inflammatory drugs (NSAIDs), ACE-inhibitors, diuretics or lithium

^ Normally distributed continuous variables, given as mean (SD) with *P* values derived from *t* tests. All other continuous variables are not normally distributed and given as median (range) with *P* values derived from Mann–Whitney *U* tests. Nominal variables are given as number (percentage)

On admission, a concomitant infection was suspected in 64 patients and proven in 31 patients. Fourteen of these concerned a bacterial infection, of which five were in the AKI group (two with a pneumonia and three with an urinary tract infection). One of these had a positive blood culture with an *Escherichia coli.* All other blood cultures remained negative.

### Deterioration of kidney function during admission

In 27 (69 %) of the 39 patients with AKI, kidney injury was already present at presentation; in the other twelve the initial KDIGO stage was 0. A deterioration of kidney function, defined as a progression of KDIGO stage during admission, was seen in 17 (44 %) of the 39 AKI patients. Eight patients eventually needed RRT; one of these patients had a normal renal function at initial presentation (Table 2). The maximal creatinine level was reached after a median of 0.3 days (range 0–3.4 days) in non-RRT patients. In the 8 RRT patients, creatinine levels rose maximally with 414 μmol (median 209 μmol) after a median of 1.3 days (range 0–3.4 days) before they were referred for dialysis.

**Table 2:** KDIGO score at initial presentation in relation to worst score during admission

	Worst KDIGO stage				
	0	1	2	3	Total
*Initial KDIGO stage*					
0	446	8	2	2	458
1		9	3	0	12
2			6	2	8
3				7	7
Total	446	17	11	11	485

### KDIGO stage versus WHO defined malarial AKI

The 2014 WHO definition for renal failure, as a criteria for severe malaria, uses the serum creatinine level at a cut-off of 265 μmol/L. At initial presentation, this criterium was met by 19 of the 485 patients (4 %) or 31 % of patients with WHO defined severe malaria (19/61). Among these 19 patients are five of the eight patients (63 %) that eventually needed RRT. Using the KDIGO classification, seven of these eight patients (89 %) would have been identified; two classified as stage 2 and five as stage 3 at presentation.

### Predictors for the outcome AKI

The receiver operating characteristics (ROC) curves of the evaluated variables are available as an Additional file 6: Figure S1. Multivariate logistic regression analysis revealed that age, leucocytes and thrombocytes were independently associated with AKI. However, they all had relatively poor positive predictive values for AKI despite acceptable negative predictive values (Table 3). Creatinine and urea were not entered in the multivariate analysis, as they belong to the defining criteria for AKI.

**Table 3:** Diagnostic performance of variables that are independently associated with AKI

Variable	Age	Thrombocytes	Leukocytes	Creatinine
*P* value	0.002	0.005	0.005	Reference
Cut-off	>43 years	≤32 × 10^9^/L	>6.1 × 10^9^/L	120 µmol/L
Sensitivity	70 (54–83)	62 (45–77)	62 (45–77)	85 (70–94)
Specificity	63 (59–68)	93 (90–95)	74 (70–78)	92 (89–94)
PPV	15 (10–20)	43 (30–57)	17 (11–24)	48 (36–60)
NPV	96 (93–98)	97 (94–98)	96 (93–98)	99 (97–100)
AUROC	0.69 (0.65–0.73)	0.80 (0.77–0.84)	0.73 (0.69–0.77)	0.90 (0.88–0.93)

### Characteristics and outcome patients needing RRT

Eight patients were referred for RRT. Their median age was 52.5 (range 36–62) and 3 (38 %) were female. Half of the patients were non-immune and half partially immune. All were admitted in the ICU and received intravenous treatment. Indications for dialysis were acidosis (4/8 patients), uremia (2/8 patients) and fluid overload (2/8 patients). None of the patients was hyperkalemic. All patients met more than one of the WHO severity criteria during admission; two met two criteria, one three, two four, one five and two met nine severity criteria.

In five of the eight RRT patients (63 %) renal function recovered fully during admission, but two patients progressed to end-stage renal disease (ESRD). In one of them the renal biopsy showed severe ischemic changes and a tubulo-interstitial nephritis with advanced scarring. She switched from haemodialysis to peritoneal dialysis and eventually received a renal transplantation. In the other patient with ESRD the renal biopsy remarkably showed a collapsing glomerulopahy, for which other causes were excluded. She is currently still on haemodialysis and is being prepared for a transplantation. Her case was described in detail previously [20]. One patient died of a suspected central pulmonary embolism 2 weeks after admission, while still on intermittent haemodialysis for the malaria-induced AKI.

## Discussion

AKI is a frequently observed complication of malaria in adults, both in malaria-endemic countries and in non-endemic regions [5–15]. The overall prevalence of AKI, defined according to the KDIGO classification, in the Rotterdam Malaria Cohort was 8 % in all patients with *P. falciparum* infection and 38 % in patients with severe disease. Age, leucocytes and thrombocytes were found to be independently associated with AKI.

Some authors report AKI incidence rates as high as 52.5 % in patients with imported malaria [15] whereas others found incidences as low as 1 % [21–23]. There are several explanations for this large variation. Most importantly, different definitions of AKI have been used. Many studies on malaria in endemic regions use the WHO criteria for severe malaria, where the serum creatinine threshold for AKI is preset at 265 μmol/L. Other studies have used the RIFLE (acronym for risk, injury, failure, loss of renal function and end stage renal disease) staging [23, 24]. In the current study the KDIGO criteria were used. The optimal serum creatinine threshold for the detection of AKI, defined as a KDIGO stage 1 or higher, was found to be 120 μmol/L, which is much lower than the threshold used in the WHO criteria. The current consensus is to define AKI according to the KDIGO criteria.

Variation in incidence rates can also be explained by differences in the patient populations that are analysed. Many studies have reported the incidence of AKI in cohorts of patients with *P. falciparum* only [10, 11, 12, 13, 14, 21, 24], while some used cohorts with malaria caused by any *Plasmodium* species [22, 23, 25, 26], and several studies report on specific subsets of patients like patients with WHO defined severe *P. falciparum* infection [10, 12, 13, 14, 24]. The immune status of the patient may also influence the risk of AKI. In previous studies it was shown that non-immune individuals with imported *P. falciparum* malaria have a higher risk of severe malaria and its complications including AKI, than partially- or semi-immune subjects [5, 6, 11, 27]. In the present study, non-immune patients were overrepresented in the AKI group.

It was previously shown that in AKI in severe malaria a return to baseline creatinine occurs after 17 ± 6 days [10]. Most patients with AKI do not require RRT and a return to baseline kidney function is achieved in the vast majority of patients in response to anti-malarial therapy and fluid resuscitation alone [5, 10, 28]. In line with these observations, only eight patients of the Rotterdam Malaria Cohort needed RRT, and five of these eight patients had a full recovery of their renal function during admission. However, the long-term prognosis of recovered AKI patients has been a matter of debate. A meta-analysis of Coca et al. [1] showed that patients with AKI are more likely to develop chronic kidney disease later on (CKD). As three of the eight RRT patients in the Rotterdam Malaria Cohort were not residents of the Netherlands and returned to their home countries not long after being discharged, information about whether or not their recovered renal functions remained stable in the long term is not available.

The most important limitation of this study is that, in the absence of data on premorbid creatinine levels, assumptions had to be made about the baseline kidney function of the included patients. Although these assumptions might be wrong in individual cases, there is no obvious reason to assume that the baseline kidney functions of the patients in this cohort, which consists mostly of international travellers with little comorbidity, are in general any different from the reference values. The method has been proposed as a suitable option in case of missing baseline renal function by the Acute Dialysis Quality Initiative (ADQI) Group and has been used before in similar studies [18, 29]. Still, the possibility remains that AKI was over-diagnosed, as some patients may have had some form of chronic kidney disease before admission with malaria. Also, information about comorbidity was absent for some patients in the database. The presence of comorbid risk factors might have contributed to the development of AKI in some patients. Another limitation is that follow-up creatinine levels were not available in the majority of patients who had a normal admission creatinine. It cannot be excluded that some of these patients developed AKI later on, but, as this concerns patients with mild disease who were discharged after a short admission, AKI is not likely to be a common complication in this group.

If AKI is detected early, measures can be taken to prevent further deterioration of kidney function and the development of associated complications. These measures include timely institution of proper parenteral anti-malarial treatment, fluid replacement, avoidance of nephrotoxic drugs and, if indicated, RRT [5, 9, 10, 28]. The use of the KDIGO classification allows for an early detection of changes in GFR, but nevertheless 12 of 39 AKI (31 %) patients in the Rotterdam Malaria Cohort had no evidence of kidney injury at first presentation. This observation emphasizes that the use of serum creatinine-based prediction models for AKI has limitations. In several animal model studies it has been shown that functional injury of the kidney, reflected in serum creatinine values, is preceded by structural injury [30]. Prediction of AKI in malaria could theoretically become more accurate with the use of markers of structural kidney injury, such as the novel biomarkers Neutrophil Gelatinase-Associated Lipocalin (NGAL) and Kidney Injury Molecule-1 (KIM-1). Recent studies, performed in a broad range of experimental and clinical settings including cardiac surgery, kidney transplantation, contrast-induced AKI and critically ill patients, show that the use of these markers may improve risk assessment [30–35]. A study in adult malaria patients in Bangladesh showed that NGAL was not superior to creatinine to predict the requirement of RRT, but patients in this study generally presented severely ill and more than half of them already had a decreased eGFR at admission [36]. One could speculate that findings in imported malaria, where patients usually tend to present much earlier in the course of the infection, could be different.

## Conclusion

Acute kidney injury is a common complication in adults with imported *P. falciparum* infection, occurring in 8 % of cases and in 38 % of the patients with severe malaria. RRT was necessary in eight of the 39 patients with AKI. The use of the KDIGO staging allows an earlier recognition of a decline in renal function in imported malaria as compared to the WHO criteria, but more research is needed to determine whether novel biomarkers can help to detect AKI in an earlier stage.

## Additional files

10.1186/s12936-015-1057-9 KDIGO criteria.
10.1186/s12936-015-1057-9 Estimated baseline serum creatinine levels.
10.1186/s12936-015-1057-9 WHO criteria for severe malaria (2014).
10.1186/s12936-015-1057-9 Descriptive statistics of diagnostic accuracy of various parameters at initial presentation for acute kidney injury (AKI).
10.1186/s12936-015-1057-9 Multivariate logistic regression analysis of predictors for outcome Acute Kidney Injury (AKI) in imported *P. falciparum* malaria.
10.1186/s12936-015-1057-9 Receiver Operating Characteristi (ROC) curves showing the ability of various parameters at initial presentation to predict development of acute kidney injury (AKI).
