# Supplementary material for: Acute kidney injury in imported Plasmodium falciparum malaria
Source: Malar J. 2015 Dec 24;14:523. doi: 10.1186/s12936-015-1057-9 (PMC4690233; doi:10.1186/s12936-015-1057-9)
Supplement: Supplementary file 1 — 10.1186/s12936-015-1057-9 KDIGO criteria. [file 12936_2015_1057_MOESM1_ESM.docx]

**Table S1.** **KDIGO criteria**

| Stage | Serum creatinine | Urine output |
| --- | --- | --- |
| 1 | 1.5–1.9 times baseline OR  ≥0.3 mg/dl (≥26.5 mmol/l) increase | < 0.5 ml/kg/h for 6–12 hours |
| 2 | 2.0–2.9 times baseline | < 0.5 ml/kg/h for ≥12 hours |
| 3 | 3.0 times baseline OR  Increase in serum creatinine to ≥4.0 mg/dl (≥353.6 mmol/l) OR  Initiation of renal replacement therapy  OR, In patients <18 years, decrease in eGFR to <35 ml/min per 1.73 m2 | <0.3 ml/kg/h for ≥24 hours OR  Anuria for ≥12 hours |
| Table adapted from [16] | | |
