# Supplementary material for: Acute kidney injury in imported Plasmodium falciparum malaria
Source: Malar J. 2015 Dec 24;14:523. doi: 10.1186/s12936-015-1057-9 (PMC4690233; doi:10.1186/s12936-015-1057-9)
Supplement: Supplementary file 2 — 10.1186/s12936-015-1057-9 Estimated baseline serum creatinine levels. [file 12936_2015_1057_MOESM2_ESM.docx]

**Table S2 .Estimated baseline serum creatinine levels**

| **Age (years)** | **Black males**  **(mg/dL [μmol/L])** | **Other males**  **(mg/dL [μmol/L])** | **Black females**  **(mg/dL [μmol/L])** | **Other females**  **(mg/dL [μmol/L])** |
| --- | --- | --- | --- | --- |
| 20–24 | 1.5 (133) | 1.3 (115) | 1.2 (106) | 1.0 (88) |
| 25–29 | 1.5 (133) | 1.2 (106) | 1.1 (97) | 1.0 (88) |
| 30–39 | 1.4 (124) | 1.2 (106) | 1.1 (97) | 0.9 (80) |
| 40–54 | 1.3 (115) | 1.1 (97) | 1.0 (88) | 0.9 (80) |
| 55–65 | 1.3 (115) | 1.1 (97) | 1.0 (88) | 0.8 (71) |
| >65 | 1.2 (106) | 1.0 (88) | 0.9 (80) | 0.8 (71) |
| Estimated glomerular filtration rate = 75 (mL/min per 1.73 m^2^) = 186 × (serum creatinine [*s*Cr]) - 1.154 × (age) - 0.203 × (0.742 if female) × (1.210 if black) = exp(5.228 - 1.154 × In [*s*Cr]) - 0.203 × In(age) - (0.299 if female) + (0.192 if black). Adapted from Bellemo *et al*. [18] | | | | |
