# Supplementary material for: Acute kidney injury in imported Plasmodium falciparum malaria
Source: Malar J. 2015 Dec 24;14:523. doi: 10.1186/s12936-015-1057-9 (PMC4690233; doi:10.1186/s12936-015-1057-9)
Supplement: Supplementary file 3 — 10.1186/s12936-015-1057-9 WHO criteria for severe malaria (2014). [file 12936_2015_1057_MOESM3_ESM.docx]

**Table S3. WHO criteria for severe malaria (2014)**

| **For epidemiological and research purposes, severe malaria is defined as one or more of the following, occurring in the absence of an identified alternative cause, and in the presence of *P. falciparum* asexual parasitemia:** | |
| --- | --- |
| **Impaired consciousness** | A Glasgow Coma Score <11 in adults or a Blantyre coma score <3 in children |
| **Acidosis** | A base deficit of >8 meq/L or, if unavailable, a plasma bicarbonate of <15 mmol/L or venous plasma lactate >5 mmol/L. Severe acidosis manifests clinically as respiratory distress – rapid, deep and laboured breathing |
| **Hypoglycemia** | Blood or plasma glucose <2.2 mmol/L (<40 mg/dL) |
| **Severe malarial anemia** | A hemoglobin concentration <5 g/dL or a hematocrit of <15% in children <12 years of age (<7 g/dL and <20%, respectively, in adults) together with a parasite count >10,000/µL |
| **Renal impairment (acute kidney injury)** | Plasma or serum creatinine >265 μmol/L (3 g/dL) or blood urea >20 mmol/L |
| **Jaundice** | Plasma or serum bilirubin >50 μmol/L (3 mg/dL) together with a parasite count >100,000/µL |
| **Pulmonary oedema** | Radiologically confirmed, or oxygen saturation <92% on room air with a respiratory rate >30/min, often with chest indrawing and crepitations on auscultation |
| **Significant bleeding** | Including recurrent or prolonged bleeding from nose gums or venepuncture sites; hematemesis or melena |
| **Shock** | Compensated shock is defined as capillary refill ≥3 sec or temperature gradient on leg (mid to proximal limb), but no hypotension. Decompensated shock is defined as systolic blood pressure <70 mm Hg in children or <80 mm Hg in adults with evidence of impaired perfusion (cool peripheries or prolonged capillary refill) |
| **Hyperparasitemia** | *P. falciparum* parasitemia >10%. A 4% parasitemia in non-immune children or adults should be considered an indicator of high risk requiring supervised management but not by itself a criterion of severe malaria |
| Table adapted from WHO 2014 criteria for severe malaria [19] | |
