# Supplementary material for: Acute kidney injury in imported Plasmodium falciparum malaria
Source: Malar J. 2015 Dec 24;14:523. doi: 10.1186/s12936-015-1057-9 (PMC4690233; doi:10.1186/s12936-015-1057-9)
Supplement: Supplementary file 4 — 10.1186/s12936-015-1057-9 Descriptive statistics of diagnostic accuracy of various parameters at initial presentation for acute kidney injury (AKI). [file 12936_2015_1057_MOESM4_ESM.docx]

| **Parameter** | **Cut-off value** | **Sensitivity** | **Specificity** | **PPV** | **NPV** | **AUROC** | **Youden** | ***P*-value*** |
| --- | --- | --- | --- | --- | --- | --- | --- | --- |
| Creatinine | >120 | 85 *(70 - 94)* | 92 *(89 - 94)* | 48 *(36- 60)* | 99 *(97-100)* | 0.90 *(0.88-0.93)* | 0.765 | reference |
| Age | >43 | 70 *(54-83)* | 63 *(59-68)* | 15 *(10-20)* | 96 *(93-98)* | 0.69 *(0.65-0.73)* | 0.332 | *<0.0001* |
| Platelets | ≤32 | 62 *(45- 77)* | 93 *(90- 95)* | 43 *(30- 57)* | 97*(94- 98)* | 0.80 *(0.77-0.84)* | 0.543 | 0.0996 |
| Leucocytes | >6.1 | 62 *(45-77)* | 74 *(70-78)* | 17 *(11-24)* | 96 *(93-98)* | 0.73 *(0.69-0.77)* | 0.354 | *0.0004* |
| CRP | >141 | 78 *(62 - 90)* | 74 *(69-78)* | 20 *(14-28)* | 98 *(95- 99)* | 0.78 *(0.74-0.82)* | 0.519 | *0.0347* |
| Sodium | ≤130 | 56 *(40-72)* | 88 *(85-91)* | 30 *(20- 42)* | 96 *(93- 98)* | 0.74 *(0.70-0.78)* | 0.450 | *0.0125* |
| Bilirubin | >35 | 76 *(59 - 88)* | 79 *(75 - 83)* | 24 *(17 - 33)* | 97 *(95 - 99)* | 0.81 *(0.78-0.85)* | 0.550 | 0.4778 |
| Urea | >6.8 | 90 *(76 - 97)* | 83 *(79 - 87)* | 32 *(24 - 42)* | 99 *(97-100)* | 0.91 (0.88-0.93) | 0.729 | 0.8990 |
| Lactate | >1.6 | 94 *(79 - 99)* | 21 *(14 - 28)* | 21 *(14 - 28)* | 99 *(96-100)* | 0.85 *(0.81-0.89)* | 0.561 | 0.4778 |
| LDH | >338 | 78 *(61 - 90)* | 74 *(70 - 78)* | 20 *(14 - 27)* | 98 *(95 - 99)* | 0.81 *(0.78-0.85)* | 0.518 | *0.0437* |
| ALAT | >49 | 76 *(60-89)* | 69 *(64-73)* | 17 *(12- 24)* | 97 *(95- 99)* | 0.74 *(0.70-0.78)* | 0.449 | *0.0110* |
| ASAT | >58 | 76 *(59- 88)* | 79 *(75- 83)* | 23 *(16- 32)* | 98 *(9 - 99)* | 0.81 *(0.77-0.84)* | 0.548 | 0.1228 |
| Parasitemia | >101,400 | 59 *(42-74)* | 84 *(80-87)* | 25 *(16-34)* | 96 *(93-98)* | 0.74 (0.70-0.78) | 0.429 | *0.0101* |
| Data are given as mean (95% confidence interval). PPV = positive predictive value, NPV = negative predictive value, AUROC = Area Under the ROC curve. Youden = Youden’s index. ***P-values of pair-wise comparison of Area Under ROC curves are given (with creatinine ROC curve as comparator). | | | | | | | | |

**Table S4. Descriptive statistics of diagnostic accuracy of various parameters at initial presentation for acute kidney injury (AKI)**
