# Supplementary material for: Acute kidney injury in imported Plasmodium falciparum malaria
Source: Malar J. 2015 Dec 24;14:523. doi: 10.1186/s12936-015-1057-9 (PMC4690233; doi:10.1186/s12936-015-1057-9)
Supplement: Supplementary file 5 — 10.1186/s12936-015-1057-9 Multivariate logistic regression analysis of predictors for outcome Acute Kidney Injury (AKI) in imported P. falciparum malaria. [file 12936_2015_1057_MOESM5_ESM.docx]

**Table S5. Multivariate logistic regression analysis of predictors for outcome Acute Kidney Injury (AKI) in imported *P. falciparum* malaria**

| **Variable** | | **P-value** | **(CI 95%)**  **Odds ratio** |
| --- | --- | --- | --- |
| **Age** | ***years*** | **0.002** | **1.076 (1.028-1.127)** |
| Immunity | *Non- or partial* | 0.357 | 0.837 (0.573-1.223) |
| Systolic blood pressure | *mm Hg* | 0.336 | 0.987 (0.961-1.014) |
| Pulse rate | *beats/min* | 0.182 | 1.022 (0.990-1.056) |
| Glasgow coma score | *EMV score* | 0.091 | 0.576 (0.304-1.093) |
| **Thrombocytes** | ***x 10⁹/L*** | **0.005** | **0.976 (0.959-0.993)** |
| **Leucocytes** | ***x 10⁹/L*** | **0.005** | **1.396 (1.106-1.762)** |
| CRP | *mg/L* | 0.876 | 1.001 (0.993-1.008) |
| Sodium | *mmol/L* | 0.406 | 0.954 (0.852-1.067) |
| Bilirubin total | *µmol/L* | 0.275 | 1.007 (0.995-1.018) |
| LDH | *U/L* | 0.441 | 0.999 (0.996-1.002) |
| ALAT | *U/L* | 0.706 | 0.998 (0.987-1.009) |
| ASAT | *U/L* | 0.137 | 1.009 (0.997-1.020) |
| LogParasitemia | *parasites/uL* | 0.496 | 0.819 (0.461-1.456) |
| 95% CI = 95% confidence interval (CI). Significant outcomes are given in bold. | | | |
