# Supplementary material for: Acute kidney injury in imported Plasmodium falciparum malaria
Source: Malar J. 2015 Dec 24;14:523. doi: 10.1186/s12936-015-1057-9 (PMC4690233; doi:10.1186/s12936-015-1057-9)

**Figure S1.** Receiver Operating Characteristi (ROC) curves showing the ability of various parameters at initial presentation to predict development of acute kidney injury(AKI)


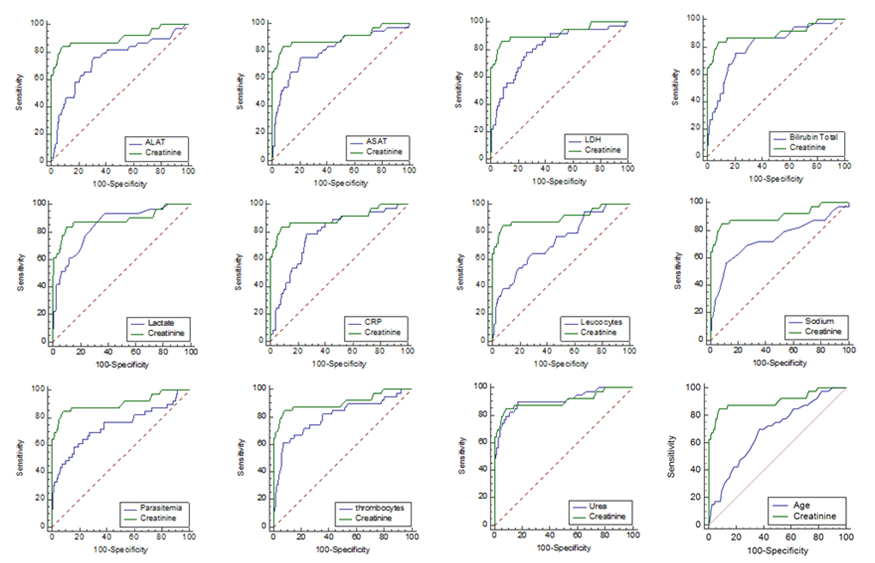

Supplement: Supplementary file 6 — 10.1186/s12936-015-1057-9 Receiver Operating Characteristi (ROC) curves showing the ability of various parameters at initial presentation to predict development of acute kidney injury (AKI). [file 12936_2015_1057_MOESM6_ESM.docx]
